# Extractable Bacterial Surface Proteins in Probiotic–Host Interaction

**DOI:** 10.3389/fmicb.2018.00645

**Published:** 2018-04-04

**Authors:** Fillipe L. R. do Carmo, Houem Rabah, Rodrigo D. De Oliveira Carvalho, Floriane Gaucher, Barbara F. Cordeiro, Sara H. da Silva, Yves Le Loir, Vasco Azevedo, Gwénaël Jan

**Affiliations:** ^1^Instituto de Ciências Biológicas, Universidade Federal de Minas Gerais (UFMG), Belo Horizonte, Brazil; ^2^STLO, Agrocampus Ouest, INRA, Rennes, France; ^3^Pôle Agronomique Ouest, Rennes, France; ^4^Bioprox, Levallois-Perret, France

**Keywords:** surface layer protein, probiotic, immunomodulation, host, adhesion

## Abstract

Some Gram-positive bacteria, including probiotic ones, are covered with an external proteinaceous layer called a surface-layer. Described as a paracrystalline layer and formed by the self-assembly of a surface-layer-protein (Slp), this optional structure is peculiar. The surface layer *per se* is conserved and encountered in many prokaryotes. However, the sequence of the corresponding Slp protein is highly variable among bacterial species, or even among strains of the same species. Other proteins, including surface layer associated proteins (SLAPs), and other non-covalently surface-bound proteins may also be extracted with this surface structure. They can be involved a various functions. In probiotic Gram-positives, they were shown by different authors and experimental approaches to play a role in key interactions with the host. Depending on the species, and sometime on the strain, they can be involved in stress tolerance, in survival within the host digestive tract, in adhesion to host cells or mucus, or in the modulation of intestinal inflammation. Future trends include the valorization of their properties in the formation of nanoparticles, coating and encapsulation, and in the development of new vaccines.

## Introduction

Probiotics are live microorganisms, traditionally regarded as safe for human consumption that, when ingested in sufficient numbers, confer a health benefit to the host (FAO/WHO, 2006). Probiotic microorganisms comprise mainly Gram-positive bacteria including LAB, bifidobacteria, enterococci, and propionibacteria. Some yeasts and Gram-negative bacteria may also be considered for probiotic use. Potential applications of probiotics involve the prevention and treatment of diarrhea caused by rotavirus, allergy and eczema, IBD; and the improvement of intestinal comfort, lactose intolerance, infection by *Helicobacter pylori*, and metabolic diseases (Syngai et al., 2016; Evivie et al., 2017). LAB constitute a large family of Gram-positive bacteria which are extensively implemented in the fermentation of a wide variety of food products. They include a variety of probiotic species: *Lactobacillus brevis*, *L. bulgaricus*, *L. plantarum*, *L. rhamnosus*, *L. casei*, *L. helveticus, L. salivarius*, *L. reuteri*, *L. johnsonii, L. fermentum*, and *L. acidophilus* (Avall-Jääskeläinen and Palva, 2005). Propionibacteria, in particular *Propionibacterium freudenreichii* strains, are emergent probiotics, also used as ripening starter in Emmental cheese manufacturing, and as vitamins producers. These propionibacteria recently revealed potent beneficial effects, including the modulation of colon cancer cells proliferation and of colon inflammation (Rabah et al., 2017). Several molecular mechanisms behind these probiotics’ beneficial effects are being elucidated. They involve modulation of the gut microbiota composition, stimulation of the epithelial barrier function, and induction of immune responses (Lebeer et al., 2008; Rabah et al., 2017). In addition, the role of bacterial surface compounds of Gram-positive bacteria includes the modulation of the gut immune system firstly, and then the systemic immune system, by mediating a cross-talk between the host and bacteria, whether they are commensals or probiotics. Such bacterial surface compounds constitute MAMPs; such as proteins, glycoproteins, lipoproteins, lipoteichoic acids, lipopolysaccharides and flagellins, which interact with the host PRRs, resulting in immune system modulation. Recently, several studies revealed the key role of surface-bound proteins, which are non-covalently attached to the cell wall, and are optionally present in certain probiotic bacteria. The surface-bound proteins may belong to a Slp lattice, an outermost macromolecular monolayer. First described in 1953 by Houwink, it consists of a paracrystalline bidimensional array made up of a Slp, which was first found on *Spirillum* sp. cell surface (Houwink, 1953; Sleytr et al., 2014). Slps are extracted using chaotropic agents such as guanidine chloride and lithium chloride (Koval and Murray, 1984). These agents may also extract other proteins, either associated to the S-layer lattice, or anchored to the cell wall through non-covalent interaction domains. These proteins include CWBDs, lysin motif domain (LysM), GW modules or SLH domains (Desvaux et al., 2006). Several studies revealed the involvement of surface-bound proteins in the bacteria/host interaction, leading to beneficial effects such as immune modulation, but the molecular mechanisms are still not fully understood. Indeed, they fulfill various crucial functions in bacteria, such as contribution to determination or maintenance of cell shape, molecular sieve, enzyme activities, contribution to adhesion, coaggregation, modulation of gut immune cells, protection against environmental stresses and antimicrobial peptides (Hynönen and Palva, 2013). The purpose of this review is to discuss involvement of non-covalently surface-bound proteins in Gram-positive probiotics’ functionalities and thus in their beneficial effects, and their future biotechnological applications.

## Occurrence, Location, and Structure of S-Layer Proteins

### S-Layer Proteins

S-layers are present in Archaea, Gram-positive and Gram-negative bacteria (Sára and Sleytr, 1996, 2000), they exhibit a thickness of 5–25 nm (Sára and Sleytr, 1996, 2000) and are highly porous (Sára and Sleytr, 1996; Sleytr and Beveridge, 1999). The S-layer paracrystalline lattice can be organized in different symmetry: oblique (p1, p2), tetragonal (p4), or hexagonal (p3, p6) symmetry (Lortal et al., 1993; Sleytr, 1997; Sleytr and Beveridge, 1999; Mobili et al., 2010). In Gram-positive bacteria, the S-layer lattice is generally composed of a single protein (Fagan and Fairweather, 2014; Pum and Sleytr, 2014; Sleytr et al., 2014), and is attached to peptidoglycan-bound SCWPs by non-covalent interactions (Fagan and Fairweather, 2014; Sleytr et al., 2014). The non-covalent anchorage of Slps may be mediated by different modules (Fagan and Fairweather, 2014). Three SLH domains can fold into a pseudo-trimer and cooperate in the binding to SCWPs. This is the most widely distributed anchorage of Slps, found in many *Bacillus* species and in the probiotic *Propionibacterium freudenreichii* (Le Maréchal et al., 2015). Another conserved anchorage mechanism is mediated via three modules of cell-wall binding domain 2 (CWB2), found in many *Clostridium* species, and binding to cell wall compounds that are still not fully elucidated (Fagan and Fairweather, 2014). By contrast, Slps from members of the *Lactobacillus* species are devoid of such motif and are anchored by a conserved CWBD, which can be C-terminal (*L. acidophilus*, *L. crispatus*) or N-terminal (*L. brevis*), while the opposite part of the protein, more variable, is involved in the self-assembly (Hynönen and Palva, 2013). The *L. acidophilus* SlpA C-terminal binding domain, which represents one-third of the protein, interacts with negatively charged SCWPs and with neutral polysaccharides (Sleytr et al., 2014).

**FIGURE 1:**
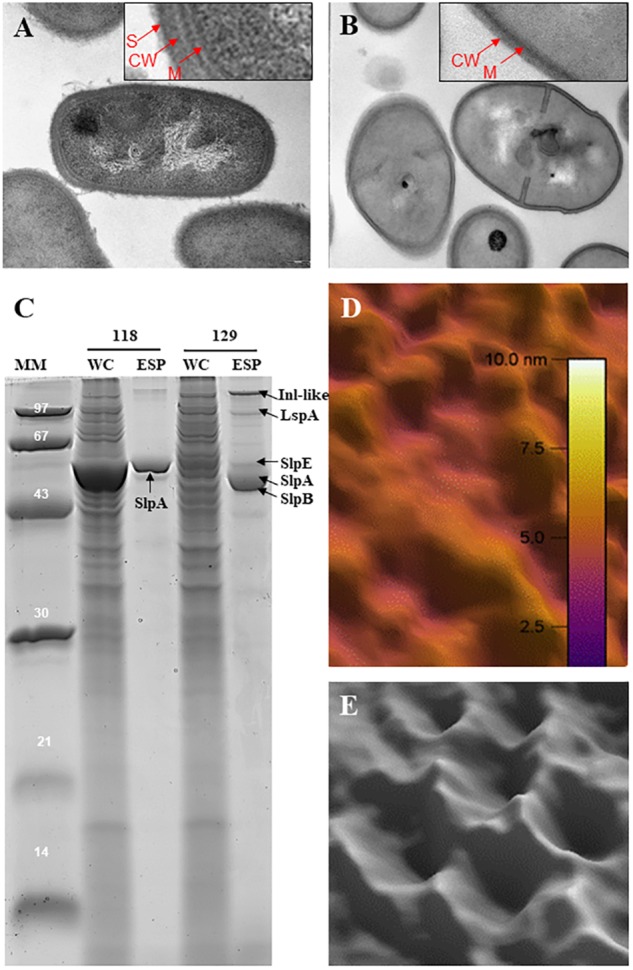
Occurrence of an S-layer is strain-dependent in *Propionibacterium freudenreichii. P. freudenreichii* CIRM-BIA 118 is covered by an outermost surface layer **(A)** that is removed by extraction using the chaotropic agent guanidine **(B)**. Red arrows indicate the membrane (M), cell wall (CW), and S-layer (S). The CIRM-BIA 118 guanidine extract was analyzed by SDS-PAGE **(C)**, showing a major band close to 58 kDa corresponding to the S-layer protein A, as identified by MS/MS (Le Maréchal et al., 2015). Extracted SlpA was dialyzed against HEPES/NaCl buffer and deposited on mica and recrystallized prior to atomic force microscopy imaging (de sa Peixoto et al., 2015). **(D)** Typical amplitude image obtained with purified Slp. **(E)** Close-up view of a Gaussian-filtered 40 nm × 40 nm phase image of recrystallized Slp showing a hexagonal arrangement. By contrast, *P. freudenreichii* CIRM-BIA 129 does not exhibit this S-layer (data not shown). However, extractable surface proteins **(C)**, in this strain, include Inl-like protein, 145 kDa, LspA (96 kDa), SlpE (59 kDa), SlpA (58 kDa), and SlpB (56 kDa). MM, molecular mass markers; WC, whole-cell SDS protein extract; ESP, extractable surface proteins guanidine extract.

**FIGURE 2:**
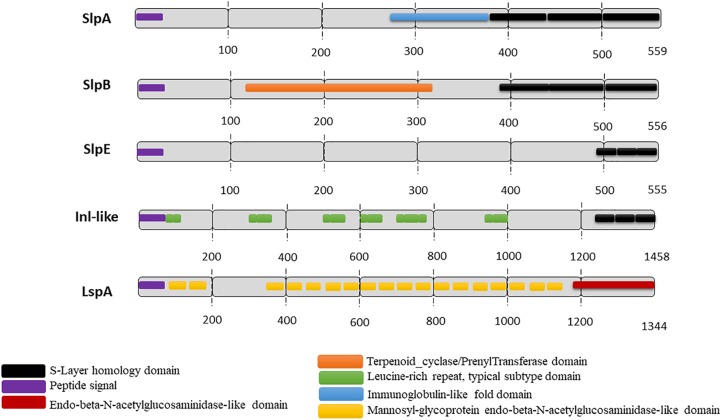
Predicted functional domains in *P. freudenreichii* extractable surface proteins. The functional domains were predicted using InterPro (EMBI/NCBI) for the five extractable surface proteins identified in different *P. freudenreichii* strains (Le Maréchal et al., 2015). S-layer protein A (SlpA), S-layer protein B (SlpB), S-layer protein E (SlpE) and Internaline-like (Inl-like) all exhibit three C-terminal conserved SLH domains allowing interaction with the peptidoglycan cell wall. By contrast, LspA exhibits no SLH domain, but *N*-acetylglucosaminidase-like domains suggesting a role in peptidoglycan metabolism.

Slps possess a molecular weight ranging from 25 to 200 kDa (Avall-Jääskeläinen and Palva, 2005) and are typically rich in acidic and hydrophobic amino acids (Sára and Sleytr, 1996, 2000; Sleytr and Beveridge, 1999; Pum and Sleytr, 2014), exhibiting a generally low isoelectric point (pI), with the exception of *Lactobacillus* Slps which have a high pI. In *P. freudenreichii*, five extractable surface proteins were identified using guanidine: SlpA, SlpB, SlpE, Internaline A (Inl A) and Large surface protein A (lsp A) (Le Maréchal et al., 2015; Deutsch et al., 2017). All these surface proteins are expressed quantitatively and qualitatively differently between different strains (Deutsch et al., 2017). However, only SlpA (illustrated in **Figure 1**) is considered as a true S-layer protein since its high expression level leads to the self-assembly of a SlpA-composed surface layer in *P. freudenreichii* strains CIRM-BIA 118 and CIRM-BIA 508 (alias CNRZ 722) (Lortal et al., 1993; de sa Peixoto et al., 2015). The SlpB protein, also presenting three SLH domains in its C-terminal domain (**Figure 2**), constitutes the major extractable surface protein in other *P. freudenreichii* strains, including CIRM BIA 129 (Le Maréchal et al., 2015). This suggests SlpB is a true S-layer protein, although the occurrence of a surface paracrystalline layer was not evidenced in these strains.

Glycosylation is the major covalent modification observed in Slps from Gram-positive bacteria. It was previously reported in *L. kefiri* and *L. buchneri* (Mobili et al., 2010). The glycosylation rate in Gram-positive bacteria Slps, leading to modification of 2–4 amino acid residues, is much lower than in Archaea. SlpB of *L. buchneri* shows four glycosylation sites consisting in *O*-glycosylation of seven glucose residues (Anzengruber et al., 2014). Slps glycosylations may be *N*- or *O*-anchored to the peptide skeleton and consist in about 50 identical units containing neutral hexoses, pentoses, heptoses or deoxyhexose and amino sugars. The Slp of *L. kefiri* is O- and N-glycosylated, with 5-8 glucose units carrying galacturonic acid (Cavallero et al., 2017). Little is known about structure–function relationships of S-layer glycan moieties (Messner et al., 2008; Schuster and Sleytr, 2015). These covalent modifications, however, may be critical for the cross-talk between bacteria carrying Slps and the host through PRRs, as demonstrated for *L. kefiri* (Prado Acosta et al., 2016).

### Other Extractable Surface-Bound Proteins

Being non-covalently anchored to the cell wall, surface-bound proteins are extracted from intact bacteria by the action of chaotropic agents such as lithium chloride and guanidine chloride. Thus, bacterial strains that do possess a true S-layer are characterized by the fact that extraction leads to the isolation of one single molecular protein species, able to re-assemble into a characteristic lattice. However, a thorough proteomic study of this extracted fraction evidenced other proteins, in addition to Slps, in *L. acidophilus*, for example, showing that Slps constitute an anchor for several other extractable surface-bound proteins called SLAPs (Johnson et al., 2013). Such proteins, identified in *Lactobacillus* species, have different functions, including interaction with the host (Johnson et al., 2013, 2016, 2017; Waśko et al., 2014; Zhang et al., 2016). In the absence of an S-layer, other non-covalently surface-bound proteins may exist and be extracted using chaotropic agents. They present different anchorage domains including SLH domains (Desvaux et al., 2006).

Surface proteome analysis of many *P. freudenreichii* strains revealed the presence of two other proteins, SlpE and Inl-like, a protein showing homology with InlA (internalin A), which exhibit C-terminal SLH domains, with a lower level of expression (**Figures 1**, **2**). They are detected in strains with or without a true SlpA surface-layer, suggesting that they are not true Slps. Regarding the protein lspA (large surface protein A), it is predicted to have a mannosyl-glycoprotein endo-beta-*N*-acetylglucosamidase-like domain and no SLH domain. Similarly, several reports further evidenced extractable surface-bound proteins in probiotic lactobacilli, and designated them as Slps, based on the presence of SLH domains, although these proteins were not shown to constitute a true paracrystalline thick surface layer. These last were, however, taken into consideration on the present review, provided that they play a role in probiotic/host interaction.

## Probiotic–Host Interaction Via Extractable Surface Proteins

The interaction between probiotic surface components and host cells may lead to modulation of gut functions (Velasquez-Manoff, 2015). Commensal bacteria colonizing the gut have co-evolved with their host and developed molecular interaction mechanisms involved in adherence, epithelial barrier function and in immune system development (Zaneveld et al., 2008; Vindigni et al., 2016). Therefore, immune cells and IECs are able to recognize several surface components (MAMPs) of autochthonous microbiota members, including lactobacilli and bifidobacteria, but also of allochthonous (food-borne) bacteria including lactobacilli, lactococci, and propionibacteria (Carvalho et al., 2017; Rook et al., 2017). The beneficial effect of probiotic bacteria, including activation of receptor-dependent pathways, is most probably favored by its ability to adhere to target cells. In this context, PRRs, including TLRs, expressed by enterocytes, are able to recognize MAMP, including extractable surface proteins.

### Extractable Surface Proteins Are Involved in Adhesion to Epithelial Cells and Extracellular Matrix Proteins

In order to exert a beneficial effect on the host, probiotic bacteria must have the ability to tolerate digestive stresses and interact with host cells [Food and Agriculture Organization of the United Nations and World Health Organization (FAO/WHO), 2002]. The adhesion of probiotic bacteria allows extending their persistence in the digestive tract, thus favoring a probiotic action. To understand how cell surface compounds from both partners can contribute to bacteria/cell adhesion, *in vitro* assays have been extensively used. This led to the development of recognized *in vitro* techniques (Blum et al., 1999; Vesterlund et al., 2005). This includes the use of IECs lines such as Caco-2 (Hirakata et al., 1998) and HT-29 (Maoret et al., 1989; Gagnon et al., 2013; Martínez-Maqueda et al., 2015). Extracellular components are also used for adhesion, including laminin, fibronectin, collagen, and proteoglycan. Intestinal mucus, an extracellular matrix composed of large glycoproteins (mucins), water, electrolyte, produced by goblet cells, may also be used. Extracellular components are reported to play a major role in modulating adhesion of microorganisms to epithelial surface (Otte and Podolsky, 2004; Johansson et al., 2011). Adhesion of probiotics to the gut mucosa may result in reduced colonization by pathogens, via competitive exclusion.

Several *in vitro* studies evidenced the involvement of extractable surface proteins, including Slps, in probiotic lactobacilli adhesion to mucus, and also to IECs. Gene inactivation of Slp genes was used in this purpose. Indeed, in *Lactobacillus acidophilus* NCFM, a knock-out mutant of the main S-layer protein, SlpA, evidenced its central role in adhesion to DCs and to their DC-SIGN receptors (Konstantinov et al., 2008). Inactivation of this gene also leads to reduced adhesion to cultured IEC (Buck et al., 2005). In the same strain, a key role of several surface layer associated proteins in adhesion was confirmed when the mutation of AcmB (β-*N*-acetylglucosaminidase) (Johnson et al., 2016), and of APF (Goh and Klaenhammer, 2010), led to a reduced binding to mucin, laminin and collagen and to IECs. By contrast, the deletion of the SLAP Serine Protease Homolog PrtX in *L. acidophilus* NCFM increases adhesion to mucin and fibronectin, which may result from the liberation of binding sites from the S-layer proteinaceous matrix (Johnson et al., 2017). In *L. salivarius* REN, inactivation of cbpA, encoding a SLAP choline-binding protein A, showed reduced adhesion to cultured IEC (Wang et al., 2017). In other strains of *L. acidophilus*, the high expression of SlpA was correlated with a high capacity to adhere to Caco-2 cells (Ashida et al., 2011). However, it was reported that mutation of *slp* genes have pleiotropic effects, including the loss of exposure of a variety of SLAPs, making it difficult to conclude the specific role of each protein species.

A role of Slps in adhesion was also suggested by investigations using extracted surface proteins. Indeed, surface extractable proteins from *L. acidophilus*, *L. brevis*, *L. helveticus*, and *L. kefiri* block DC-SIGN receptors *in vitro* and prevent adhesion of pathogenic bacteria to DC-SIGN expressing cells (Prado Acosta et al., 2016). Furthermore, lithium extraction of *L. acidophilus* fb214 surface proteins reduces adhesion to cultured IECs (Meng et al., 2014). In *L. acidophilus* NCFM, FbpB is a SLAP, showing a fibronectin-binding domain, which mediates adhesion to mucin and fibronectin *in vitro* (Hymes et al., 2016). Accordingly, *L. brevis* ATCC 8287 surface layer shows a high affinity to laminin and to fibronectin, and its removal affects *L. brevis* adhesion to intestinal cells and to extracellular matrix proteins (Hynönen et al., 2002; de Leeuw et al., 2006; Uroić et al., 2016). Extracted surface bound proteins from probiotic lactobacilli were shown by different authors to bind *in vitro* to host cells proteins and extracellular matrix (Chen et al., 2007; Johnson-Henry et al., 2007; Carasi et al., 2014; Waśko et al., 2014; Prado Acosta et al., 2016; Zhang et al., 2016). However, such results should be considered with care, as Slps are poorly soluble, forming aggregates in aqueous environments, which renders interpretation of results difficult.

Adhesion to IECS and to mucus was also reported for dairy propionibacteria (Cousin et al., 2012). Nonetheless, the adhesion rates were highly variable, depending on the adhesion model used, the strain and the growth conditions (Cousin et al., 2010; Rabah et al., 2017). A comparative study of *P. freudenreichii* strains, in terms of 1) adhesion rate and 2) surface proteome, led to the identification of propionibacterial SlpB as a potent adhesin, which was further confirmed by *slpb* gene inactivation (de Carmo et al., 2017). One protein, Inl-like (**Figure 2**) contains several leucine-rich repeats (LRRs), predicted to be involved in protein binding. It shows homologies with InlA, which functions as an adhesin in *Listeria monocytogenes*. However, no functional characterization was undertaken to study the role of this protein in *P. freudenreichii* adhesion. Furthermore; the S-layer protein A of *P. freudenreichii* is predicted to have an Immunoglobulin-like fold domain, found in some surface proteins in pathogen bacteria (Buts et al., 2003; Lin et al., 2010), where it is reported to play a role in adhesion to host cells.

The *in vitro* investigations reported here indicate a role of Slps and other associated proteins in adhesion to mucus components and to IECs, which is a prominent feature for probiotic bacteria to trigger beneficial effects within the gut mucosa. Nonetheless, *in vivo* studies are needed to confirm the role of Slps in adhesion, and thus in the persistence of probiotic bacteria within the gut. In addition, Slps-mediated adhesion to mucus and IECs is poorly understood, but is thought to lead to inhibition of pathogenic microorganisms adhesion (Hynönen and Palva, 2013; Sleytr et al., 2014).

### Inhibition of Pathogens by Extractable Surface Proteins

Inhibition of bacterial or viral infections is another beneficial application reported for probiotic bacteria, in which extractable surface proteins may play an important role. Indeed, adhesion of probiotic bacteria to the intestinal mucosa, via surface extractable adhesins interacting with host PRRs, may result in the inhibition of pathogens invasion by competitive exclusion. As an example, *L. helveticus* R0052 inhibits adhesion of entero-hemorrhagic *Escherichia coli* to caco-2 cells and so does its lithium surface proteins extract (Johnson-Henry et al., 2007), which coaggregates with several pathogen species (Beganović et al., 2011; Waśko et al., 2014). In addition, Slps of *L. crispatus* ZJ001 (Chen et al., 2007), *L. kefir* (Golowczyc et al., 2007), *L. salivarius*, and *L. reuteri* (Zhang et al., 2013) were proposed to be responsible for competitive exclusion of bacterial pathogens such as *E. coli* and *Salmonella* species. A similar effect was shown for *L. acidophilus* Slps which inhibited adherence and invasion of Caco-2 cells by *Salmonella enterica* serovar Typhimurium (Li et al., 2010, 2011) and protected cells by restoring the transepithelial resistance. This resistance is a recognized marker of barrier integrity of tight junctions, in epithelial cell lines (Klingberg et al., 2005). These Slps inhibited the mitogen-activated protein kinase (MAPK) signaling pathways induced by *S. typhimurium* (Li et al., 2011). In the same way, *L. plantarum* CGMCC 1258 MIMP protein, an extractable surface protein, limits pathogen invasion by inducing the expression of Claudin-1, Occludin, JAM-1, and ZO-1, by restoring tight junction damage and thus epithelium integrity (Qin et al., 2009; Liu et al., 2011a,b). Indeed, the intestinal mucosa tightness, relying on tight junctions (or “zonula occludens”), is impaired in several inflammatory and infections gut diseases (Vélez et al., 2007; Sengupta et al., 2013).

A potential role of extractable surface proteins covering *L. acidophilus* ATCC4356, *L. brevis* ATCC14869, *L. helveticus* ATCC12046 or *L. kefiri* JCM5818 against bacterial infection of cells was observed (Prado Acosta et al., 2016). Cells expressing the DC-specific DC-SIGN receptor exhibited a reduced susceptibility to bacterial infections, as a result of pre-treatment with Lactobacilli Slps. Moreover, pre-treatment of the pathogens (both Gram-negative and mycobacterial models) with Slps from *L. acidophilus* ATCC 4356 and *L. brevis* ATCC 14869 reduces pathogens viability but also prevents infection (Prado Acosta et al., 2016). DC-SIGN is a CLR present in DCs and macrophages, it is involved in the recognition of several viruses and other microbes, a key step in the entry of pathogens into the cell. Indeed, *L. acidophilus* ATCC4356 Slps stimulate the activation of mouse DCs by activating IFN-I signaling pathway, which inhibit invasion of DCs by influenza virus H9N2 (Gao et al., 2016). In addition, *L. acidophilus* ATCC4356 Slps inhibit the Junin virus (JUNV) infection by interacting with DC-SIGN (Martínez et al., 2012).

These data suggest a role of probiotics extractable surface proteins, including Slps, in the prevention of host colonization by pathogens responsible for infectious diarrhea. Once again, data obtained using extracted surface bound proteins should be considered with care and these hypotheses should be confirmed *in vivo*.

### Immunomodulatory Properties of Extractable Surface Proteins

**FIGURE 3:**
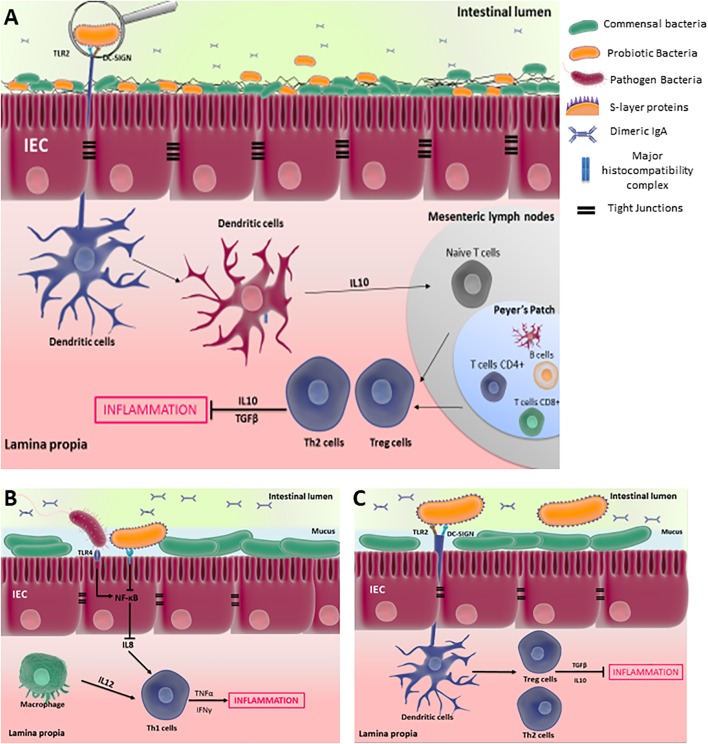
Cross-talk between probiotic bacteria and the host, mediated by IECs and immunes cells, within the gut associated lymphoid tissues (GALT). **(A)** An overview of the interaction of antigen-presenting-cells such as DCs with probiotic bacterian, which initiates a tolerance response by inducing Treg/Th2 anti-inflammatory response; while DCs-pathogenic bacteria interaction induces a Th1/Th17 proinflammatory response. **(B)** S-layer proteins inhibit the proinflammatory response of epithelial cells by reducing NF-κB activity, which is induced by pathogenic bacteria; **(C)** S-layer proteins are recognized by DCs via DC-SIGN and TLR2 receptors, inducing tolerance response in the GALT. These hypothetical schemata are mainly based on *in vitro* investigations.

Selected strains of LAB, including *L. acidophilus*, and of PROPIONIBACTERIA, including *P. freudenreichii*, exert anti-inflammatory properties in the context of colitis, by modulating gut immunity. Intestinal homeostasis is tightly governed by regulatory immune mechanisms, which are established by interactions involving commensal/probiotic bacteria and host PRRs, including CLRs and TLRs. The disruption of such regulatory mechanisms may result in IBD. **Figure 3A** illustrates the cross-talk between probiotic bacteria and the host, mediated by IECs and immunes cells within the GALT, which initiates an immune responses according to the MAMPs recognized by various PRRs. This hypothetical schema is mainly based on *in vitro* investigations.

Detailed studies revealed the crucial role of Slps in host–probiotic interactions mediated by intestinal cells, which are an important protagonist at the forefront to maintain gut immunity homeostasis. *L. helveticus* MIMLh5 anti-inflammatory effects on Caco-2 cells is mediated by its SlpA and reduces activation of NF-κB (Taverniti et al., 2013). *L. acidophilus* contains three different Slps, SlpA, SlpB, and SlpX, which interact with PRRs and modulate the immune response. *L. acidophilus* Slps decrease interleukin (IL) 8 secretion in Caco-2 cells stimulated by *S. typhimurium* (Li et al., 2011). IL-8 cytokine is an important proinflammatory mediator secreted by intestinal cells, as well as by activated macrophages, leading, in synergy with IL-12, to the development of T helper (Th1) cells in the intestinal mucosa (Sanchez-Muñoz et al., 2008). **Figure 3B** illustrates how probiotics, via the recognition of Slps, may reduce activation of NF-κB and therefore expression of IL8 in IECs, limiting the proinflammatory response induced by pathogen.

Besides the interaction with IECs, Slps interact with antigen-presenting cells such as DCs, which reside in the Peyer’s patch, lamina propria and mesenteric lymph nodes. As schematized in **Figure 3A**, DCs are the main stimulators of naive T cells, which distinguishes them from all other antigen presenting cells. Depending on the microbial stimulus encountered, DCs promote the differentiation of naïve T cells toward Th1, Th2, unpolarized T cells, Th17 or T regulatory cell responses. Investigation of the role of *L. acidophilus* Slps provided insights into immune cells-Slps interactions and the resulting immune response within the gut. The high expression of SlpA in *L. acidophilus* L92 was correlated with high induction of IL-12p70 secretion during splenocytes stimulation (Ashida et al., 2011). However, SlpA of *L. acidophilus* was reported to confer anti-inflammatory traits to the strain. Indeed, mutation of *L. acidophilus* NCFM SlpA results in a chromosomal inversion leading to dominant expression of SlpB. This mutant induces higher levels of proinflammatory cytokines such as IL-12p70, tumor necrosis factor-α (TNFα), and IL-1, compared to the wild-type strain, in DCs. However, both SlpA and SlpB activated TLR-2 at similar levels, an interaction that appeared to be crucial for the activating of IL-4-producing T cells (Konstantinov et al., 2008). The protective role of SlpA in the context of colitis was further elucidated. *L. acidophilus* NCK2187, which solely expresses SlpA, and its purified SlpA, both bind to the C-type lectin SIGNR3 to induce regulatory signals that result in alleviation of colitis (Lightfoot et al., 2015). However, such protection was not observed in Signr3-/- mice, suggesting that the SlpA/SIGNR3 interaction plays a key regulatory role in the healing of colitis (Lightfoot et al., 2015). Other extractable surface proteins are involved in *L. acidophilus* NCFM immunomodulatory properties (Johnson et al., 2013, 2017). Indeed, mutation of the S-layer-associated serine protease homolog (Prtx) in *L. acidophilus* NCFM enhanced stimulation of IL-6, IL-12, and IL-10, compared to wild-type, when exposed to mouse DCs (Johnson et al., 2017). The authors suggest that PrtX may degrade certain cytokines, so that amounts of cytokines are higher when using the mutant. Mutation of Lba-1029, a putative SLAP in *L. acidophilus* NCFM, revealed its role in a pro-inflammatory response in murine DCs, including TNFα secretion (Johnson et al., 2013). SLAPs may thus take part in probiotics immunological properties. Slps of *L. helveticus* NS8, isolated from fermented koumiss, had no effect on the basal production of IL-10 in mouse macrophage cell line RAW264.7, but decreased IL12 expression triggered by LPS stimulation, suggesting an anti-inflammatory potential (Rong et al., 2015). Lactobacilli Slps contribute to the anti-inflammatory effect of probiotic bacteria within GALT, by interacting with DCs via different PRRs, which initiate the differentiation of Treg cells as illustrated by **Figure 3C**. Contrastingly, *L. helveticus* MIMLh5 and its SlpA act as stimulators of the innate immune system by triggering the expression of proinflammatory mediators such as TNFα and cyclooxygenase 2 (COX-2) in the human macrophage cell line U937 via TLR2 recognition (Taverniti et al., 2013). In the same experiments, this purified SlpA did not affect the expression of the anti-inflammatory cytokine interleukin-10 (Taverniti et al., 2013). Similarly, *L. brevis* Slps induce TNFα production in monocyte-derived dendritic cells (moDCs) (Uroić et al., 2016).

Immunomodulation was also reported for PROPIONIBACTERIA. As an example, strain-dependent immunomodulatory properties were evidenced *in vitro* using human PBMCs (Foligné et al., 2013; Deutsch et al., 2017) and then confirmed *in vivo* in a mouse colitis model (Foligné et al., 2010). Extractable surface proteins were involved in this modulatory effect (Le Maréchal et al., 2015). Indeed, the extracted proteins induce regulatory IL-10, in a dose-dependent manner, in PBMCs, with little or no secretion of pro-inflammatory factors (IL-12, TNFα, and IL6) (Le Maréchal et al., 2015). In addition, they reduce the proinflammatory response triggered by the proinflammatory strain *Lactococcus lactis* MG1363 in PBMCs (Le Maréchal et al., 2015). Inactivation of the gene encoding SlpB suppress IL-10 induction by *P. freudenreichii*, and so does inactivation of SlpE (Deutsch et al., 2017). In the same time, expression of other surface proteins, including SlpF and moonlighting proteins, is also correlated with this anti-inflammatory trait in *P. freudenreichii*. By contrast, *P. freudenreichii* strains expressing high level of SlpA, a true S-layer protein, exert no immunomodulatory effect. The authors suggest that the immunomodulatory properties of *P. freudenreichii* strains result from a combination of several surface proteins (Deutsch et al., 2017). By contrast with lactobacilli, mechanisms involved in the interaction between PRRs and PROPIONIBACTERIA surface proteins remain unknown.

In conclusion, probiotic bacteria, via Slps, may have an immunomodulatory effect mediated by C-type lectin and TLR receptors within GALT. However, the effective role of these proteins should be confirmed *in vivo* in order to give tools to fight gut inflammation.

## Protective Role of Probiotics’ Extractable Surface Proteins

Bacterial surface layers are generally recognized as the outermost structure of the bacterial cell (Gerbino et al., 2015a). They are thus widely considered to play a key role as an interface between intra- and extra-cellular compartments, and thus between the bacterium and its environment or its host. This interface was most likely developed as a consequence of the selective pressure generated by these interactions. They may act as a physical barrier against external factors (Sleytr and Messner, 1988), and also prevent the release of cellular molecules (Sleytr and Messner, 1988). Indeed, they are described as a molecular sieve, forming a highly porous structure with pores exhibiting identical morphology and size, within a bacterial strain, with some variations among strains. The porosity of this layer occupies a surface area that can go up to 70% (Sleytr et al., 2001; Avall-Jääskeläinen and Palva, 2005). Studies on permeability have shown that some S-layers prevent the entry of molecules with molecular weights exceeding 10,000–15,000 kDa, providing the strain with a selective advantage (Lortal et al., 1993).

The presence of surface layers was reportedly linked with enhanced tolerance toward stresses. Presence of an S-layer was reported to decrease *L. helveticus* susceptibility to mutanolysin (Lortal et al., 1992). S-layers were furthermore shown to resist to extreme environmental conditions, even in extremophile bacteria, and to digestive assaults including variations in pH, bile salts, proteases and simulated gastrointestinal conditions (Smit et al., 2001; Chen et al., 2007; Eslami et al., 2013). In accordance, coating of liposomes with S-layer proteins from *L. brevis* and from *L. kefiri* increased their stability upon exposure to bile salts, pancreatic extract and pH, as compared to uncoated liposomes (Hollmann et al., 2007). On the other hand, removal of Slps caused enhanced *L. hilgardii* susceptibility toward bacteriolytic enzymes and physicochemical stress (Dohm et al., 2011). Furthermore, removal of the surface layer using the chaotropic agent lithium chloride drastically affects survival of *L. acidophilus* and of *L. helveticus* in simulated gastric and intestinal conditions (Frece et al., 2005; Meng et al., 2014). In accordance with a physiological role of S-layer proteins in defense mechanisms, their expression is induced by stimuli participating in digestive stress. In *L. acidophilus*, exposition to bile increases expression of SlpA in the ATCC 4356 strain (Khaleghi et al., 2010). The same authors further evidenced similar induction of this protein by acidic pH and heat stress (Khaleghi and Kasra, 2012). Among bile constituents, bile salts were further evidenced as the stimuli responsible for S-layer induction in *L. acidophilus* IBB 8001 (Grosu-Tudor et al., 2016). The induction of Slps expression may thus take part in a general strategy to adapt and survive harsh environmental conditions encountered in the environment, in the digestive tract, or in the succession thereof (Butler et al., 2013; Gerbino et al., 2015a). However, contrasting informations result from experimental gene inactivation of S-layer proteins in probiotic bacteria. Such mutations were indeed reported to drastically affect interactions with the host, including adhesion (do Carmo et al., 2017; Wang et al., 2017) and immune modulation (Konstantinov et al., 2008; Deutsch et al., 2017). However, their impact on probiotics stress tolerance is still elusive. Deletion of the *slpa* gene caused alterations in cell envelope structure and defect in resistance to solvent and shear stresses in the environmental extremophilic bacterium *Deinococcus radiodurans* (Rothfuss et al., 2006). In the probiotic *L. acidophilus*, the auxiliary S-layer protein SlpX, identified as a protein associated with the S-layer complex, plays a role in its permeability. Indeed, inactivation of SlpX affects the growth rate and the tolerance to bile salts in the NCK1962 mutant, as well as its relative overexpression in NCK1377, a SlpB-dominant strain that lacks SlpA protein. The NCK1962 slpX-negative mutant is more susceptible to SDS, yet more resistant to bile, than the wild type (Goh et al., 2009). Aggregation-promoting factors (Apf) are proteins considered as “S-layer-like” and identified at the surface of several lactobacilli. They share several characteristics with *Lactobacillus* S-layer proteins, such as their relative abundance on the cell surface, extractability by lithium chloride (LiCl), amino acid composition, predicted physical properties like high pI and indispensability for growth (Ventura et al., 2002; Jankovic et al., 2003). Mutation of the corresponding *apf* gene in *L. acidophilus* resulted in enhanced susceptibility to SDS, to bile and to intestinal and gastric juices (Goh and Klaenhammer, 2010). This suggests reduced survival during transit through the digestive tract.

Finally, S-layers may also play a role in detoxification. The biosorption of toxic compounds, including uranium (Hennig et al., 2001) and heavy metals (Velásquez and Dussan, 2009) was reported for telluric bacteria belonging to the *Bacillus* species. It was attributed to their S-layers (Merroun et al., 2005). Such heavy metal biosorption was later reported for *L. kefir* (Gerbino et al., 2015b) with a key role of S-layer proteins. Biosorption of heavy metals and of mycotoxins was reported in *L. rhamnosus* and in *P. freudenreichii* (Ibrahim et al., 2006; Halttunen et al., 2008). These last were shown in a clinical study to reduce the biologically effective dose of aflatoxin exposure and may thereby offer an effective dietary approach to decrease the risk of liver cancer (El-Nezami et al., 2006).

## Biotechnological Applications

The peculiar property of Slps to auto-assemble and to form reproducible supramolecular aggregates that are reputed irreversible and resistant to physicochemical assaults naturally led to the idea to use them in the field of (nano)biotechnology (Hynönen et al., 2002; Sleytr et al., 2011). Such monomolecular arrays provide well-defined structures, depending on the physicochemical properties of the glycoprotein, which constitutes the closed, isoporous lattice, and for which a wide biodiversity exists, among bacteria. This led to investigate the application of re-crystalized Slps to develop ultrafiltration membranes with very accurate molecular cutoffs, good stability afforded by intramolecular cross-linking, low membrane fouling and tunable surface properties in terms of net charges and hydrophilicity. Furthermore, chemical modifications and genetic engineering allow the immobilization of functional molecules, including enzymes, ligands, antigens and antibodies, while retaining the self-assembly properties of Slps. Some Slps being known to spontaneously produce preformed nanoparticles, on native surface-layers, functionalized Slps nanoparticles were made, including metallic and semiconductor nanoparticles. Other applications include Slps as supporting structures for functional lipid membranes, or for vaccine development. Indeed, conjugate vaccines with Slps and antigens, haptens or recombinant allergens, gave promising results in vaccination trials, due to intrinsic adjuvant properties of some SlpS. For a review on Slps biotechnological applications, se the review by Sleytr et al. (2014).

## Conclusion

Extractable surface proteins, with various properties, have been described in several species and strains of probiotic bacteria. The peculiar properties of extractable surface proteins, including abundant expression, self-assembly, surface location, resistance to physicochemical assaults, immunomodulation, adhesion and toxic remediation, offer the possibility to orientate the biological properties of fermented food products and of probiotic food supplements. S-layer proteins have a great potential in the field of nanobiotechnology, because of their ability to form repetitive protein arrays by spontaneous association (Hynönen et al., 2002; Sleytr et al., 2011). This applies to vaccine candidates, to surface display of epitope, of proteins with therapeutic or biotechnologic interest (Michon et al., 2016). This opens promising perspectives in the field of gut disorders, including IBS and IBD, infectious diseases, as well as oral vaccination. Future trends include engineering of Slps for specific, efficient and cost-effective targeting of desired antigens and other medically important molecules.

## Author Contributions

GJ, YLL, and VA supervised the work and corrected the manuscript. FLRdC and HR did the main part of the bibliographical survey. All the authors took part in the writing of the manuscript.

## Conflict of Interest Statement

The authors declare that the research was conducted in the absence of any commercial or financial relationships that could be construed as a potential conflict of interest.

## Abbreviations

APF: aggregation-promoting-like factor
CLR: C-type lectin receptor
CWBD: cell wall binding domain
DC: dendritic cell
DC-SIGN: dendritic cell-specific intercellular adhesion molecule-3-grabbing non-integrin
FAO: Food and Agriculture Organization
GALT: gut-associated lymphoid tissue
IBD: inflammatory bowel disease
IEC: intestinal epithelial cell
LAB: lactic acid bacteria
MAMP: microbe-associated molecular pattern
PBMC: peripheral blood mononuclear cell
PRR: pattern recognition receptor
SCWP: secondary cell-wall polymer
SLAP: S-layer associated protein
SLH: S-layer homology domain
Slp: surface-layer protein
TLR: toll-like receptor
WHO: World Health Organization

## References

[B1] Anzengruber J. Pabst M. Neumann L. Sekot G. Heinl S. Grabherr R. (2014). Protein O-glucosylation in *Lactobacillus buchneri*. *Glycoconj. J.* 31 117–131. 10.1007/s10719-013-9505-7 24162649 PMC4396861

[B2] Ashida N. Yanagihara S. Shinoda T. Yamamoto N. (2011). Characterization of adhesive molecule with affinity to Caco-2 cells in *Lactobacillus acidophilus* by proteome analysis. *J. Biosci. Bioeng.* 112 333–337. 10.1016/j.jbiosc.2011.06.001 21763196

[B3] Avall-Jääskeläinen S. Palva A. (2005). *Lactobacillus* surface layers and their applications. *FEMS Microbiol. Rev.* 29 511–529. 10.1016/j.femsre.2005.04.003 15935509

[B4] Beganović J. Frece J. Kos B. Leboš Pavunc A. Habjanič K. Sušković J. (2011). Functionality of the S-layer protein from the probiotic strain *Lactobacillus helveticus* M92. *Antonie Van Leeuwenhoek* 100 43–53. 10.1007/s10482-011-9563-4 21327475

[B5] Blum S. Reniero R. Schiffrin E. J. Crittenden R. Mattila-Sandholm T. Ouwehand A. C. (1999). Adhesion studies for probiotics: need for validation and refinement. *Trends Food Sci. Technol.* 10 405–410. 10.1016/S0924-2244(00)00028-5

[B6] Buck B. L. Altermann E. Svingerud T. Klaenhammer T. R. (2005). Functional analysis of putative adhesion factors in *Lactobacillus acidophilus* NCFM. *Appl. Environ. Microbiol.* 71 8344–8351. 10.1128/AEM.71.12.8344-8351.2005 16332821 PMC1317474

[B7] Butler È. Alsterfjord M. Olofsson T. C. Karlsson C. Malmström J. Vásquez A. (2013). Proteins of novel lactic acid bacteria from *Apis mellifera mellifera*: an insight into the production of known extra-cellular proteins during microbial stress. *BMC Microbiol.* 13:235. 10.1186/1471-2180-13-235 24148670 PMC4015849

[B8] Buts L. Bouckaert J. De Genst E. Loris R. Oscarson S. Lahmann M. (2003). The fimbrial adhesin F17-G of enterotoxigenic *Escherichia coli* has an immunoglobulin-like lectin domain that binds N-acetylglucosamine. *Mol. Microbiol.* 49 705–715. 12864853 10.1046/j.1365-2958.2003.03600.x

[B9] Carasi P. Ambrosis N. M. De Antoni G. L. Bressollier P. Urdaci M. C. Serradell M. L. Á. (2014). Adhesion properties of potentially probiotic *Lactobacillus kefiri* to gastrointestinal mucus. *J. Dairy Res.* 81 16–23. 10.1017/S0022029913000526 24168928

[B10] Carvalho R. D. Breyner N. Menezes-Garcia Z. Rodrigues N. M. Lemos L. Maioli T. U. (2017). Secretion of biologically active pancreatitis-associated protein I (PAP) by genetically modified dairy *Lactococcus lactis* NZ9000 in the prevention of intestinal mucositis. *Microb. Cell Fact.* 16:27. 10.1186/s12934-017-0624-x 28193209 PMC5307810

[B11] Cavallero G. J. Malamud M. Casabuono A. C. Serradell M. L. Á. Couto A. S. (2017). A glycoproteomic approach reveals that the S-layer glycoprotein of *Lactobacillus kefiri* CIDCA 83111 is O- and N-glycosylated. *J. Proteomics* 162 20–29. 10.1016/j.jprot.2017.04.007 28433761

[B12] Chen X. Xu J. Shuai J. Chen J. Zhang Z. Fang W. (2007). The S-layer proteins of *Lactobacillus crispatus* strain ZJ001 is responsible for competitive exclusion against *Escherichia coli* O157:H7 and *Salmonella* typhimurium. *Int. J. Food Microbiol.* 115 307–312. 10.1016/j.ijfoodmicro.2006.11.007 17289201

[B13] Cousin F. J. Deutsch S.-M. Perez Chaia A. Foligné B. Jan G. (2012). Interactions between probiotic dairy propionibacteria and the intestinal epithelium. *Curr. Immunol. Rev.* 8 216–226. 10.2174/157339512800671976

[B14] Cousin F. J. Mater D. D. G. Foligné B. Jan G. (2010). Dairy propionibacteria as human probiotics: a review of recent evidence. *Dairy Sci. Technol.* 91 1–26. 10.1051/dst/2010032

[B15] de Leeuw E. Li X. Lu W. (2006). Binding characteristics of the *Lactobacillus brevis* ATCC 8287 surface layer to extracellular matrix proteins. *FEMS Microbiol. Lett.* 260 210–215. 10.1111/j.1574-6968.2006.00313.x 16842346

[B16] de sa Peixoto P. Roiland C. Thomas D. Briard-Bion V. Le Guellec R. Parayre S. (2015). Recrystallized S-layer protein of a probiotic propionibacterium: structural and nanomechanical changes upon temperature or pH shifts probed by solid-state NMR and AFM. *Langmuir* 31 199–208. 10.1021/la503735z 25479375

[B17] Desvaux M. Dumas E. Chafsey I. Hébraud M. (2006). Protein cell surface display in Gram-positive bacteria: from single protein to macromolecular protein structure. *FEMS Microbiol. Lett.* 256 1–15. 10.1111/j.1574-6968.2006.00122.x 16487313

[B18] Deutsch S.-M. Mariadassou M. Nicolas P. Parayre S. Le Guellec R. Chuat V. (2017). Identification of proteins involved in the anti-inflammatory properties of *Propionibacterium freudenreichii* by means of a multi-strain study. *Sci. Rep.* 7:46409. 10.1038/srep46409 28406170 PMC5390290

[B19] de Carmo F. L. R. Rabah H. Huang S. Gaucher F. Deplanche M. Dutertre S. (2017). *Propionibacterium freudenreichii* surface protein SlpB is involved in adhesion to intestinal HT-29 cells. *Front. Microbiol.* 8:1033. 10.3389/fmicb.2017.01033 28642747 PMC5462946

[B20] do Carmo F. L. R. Rabah H. Huang S. Gaucher F. Deplanche M. Dutertre S. (2017). *Propionibacterium freudenreichii* surface protein SlpB is involved in adhesion to intestinal HT-29 cells. *Front. Microbiol.* 8:1033. 10.3389/fmicb.2017.01033 28642747 PMC5462946

[B21] Dohm N. Petri A. Schlander M. Schlott B. König H. Claus H. (2011). Molecular and biochemical properties of the S-layer protein from the wine bacterium *Lactobacillus hilgardii* B706. *Arch. Microbiol.* 193 251–261. 10.1007/s00203-010-0670-9 21221529

[B22] El-Nezami H. S. Polychronaki N. N. Ma J. Zhu H. Ling W. Salminen E. K. (2006). Probiotic supplementation reduces a biomarker for increased risk of liver cancer in young men from Southern China. *Am. J. Clin. Nutr.* 83 1199–1203. 16685066 10.1093/ajcn/83.5.1199

[B23] Eslami N. Kermanshahi R. K. Erfan M. (2013). Studying the stability of S-layer protein of *Lactobacillus acidophilus* ATCC 4356 in simulated gastrointestinal fluids using SDS-PAGE and circular dichroism. *Iran. J. Pharm. Res.* 12 47–56. 24250671 PMC3813368

[B24] Evivie S. E. Huo G.-C. Igene J. O. Bian X. (2017). Some current applications, limitations and future perspectives of lactic acid bacteria as probiotics. *Food Nutr. Res.* 61:1318034. 10.1080/16546628.2017.1318034 28659729 PMC5475324

[B25] Fagan R. P. Fairweather N. F. (2014). Biogenesis and functions of bacterial S-layers. *Nat. Rev. Microbiol.* 12 211–222. 10.1038/nrmicro3213 24509785

[B26] FAO/WHO (2006). *Probiotics in Food: Health and Nutritional Properties and Guidelines for Evaluation*. Report of a Joint FAO/WHO Working Group on Drafting Guidelines for the Evaluation of Probiotics in Food. Rome: FAO.

[B27] Foligné B. Breton J. Mater D. Jan G. (2013). Tracking the microbiome functionality: focus on *Propionibacterium* species. *Gut* 62 1227–1228. 10.1136/gutjnl-2012-304393 23389969

[B28] Foligné B. Deutsch S.-M. Breton J. Cousin F. J. Dewulf J. Samson M. (2010). Promising immunomodulatory effects of selected strains of dairy propionibacteria as evidenced *in vitro* and *in vivo*. *Appl. Environ. Microbiol.* 76 8259–8264. 10.1128/AEM.01976-10 20971874 PMC3008228

[B29] Food and Agriculture Organization of the United Nations and World Health Organization (FAO/WHO) (ed.) (2002). *Probiotics in Food: Health and Nutritional Properties and Guidelines for Evaluation.* Rome: Food and Agriculture Organization of the United Nations.

[B30] Frece J. Kos B. Svetec I. K. Zgaga Z. Mrsa V. Suskovic J. (2005). Importance of S-layer proteins in probiotic activity of *Lactobacillus acidophilus* M92. *J. Appl. Microbiol.* 98 285–292. 15659182 10.1111/j.1365-2672.2004.02473.x

[B31] Gagnon M. Zihler Berner A. Chervet N. Chassard C. Lacroix C. (2013). Comparison of the Caco-2, HT-29 and the mucus-secreting HT29-MTX intestinal cell models to investigate *Salmonella* adhesion and invasion. *J. Microbiol. Methods* 94 274–279. 10.1016/j.mimet.2013.06.027 23835135

[B32] Gao X. Huang L. Zhu L. Mou C. Hou Q. Yu Q. (2016). Inhibition of H9N2 virus invasion into dendritic cells by the S-Layer protein from *L. acidophilus* ATCC 4356. *Front. Cell. Infect. Microbiol.* 6:137. 10.3389/fcimb.2016.00137 27826541 PMC5078685

[B33] Gerbino E. Carasi P. Mobili P. Serradell M. A. Gómez-Zavaglia A. (2015a). Role of S-layer proteins in bacteria. *World J. Microbiol. Biotechnol.* 31 1877–1887. 10.1007/s11274-015-1952-9 26410425

[B34] Gerbino E. Carasi P. Araujo-Andrade C. Tymczyszyn E. E. Gómez-Zavaglia A. (2015b). Role of S-layer proteins in the biosorption capacity of lead by *Lactobacillus kefir*. *World J. Microbiol. Biotechnol.* 31 583–592. 10.1007/s11274-015-1812-7 25653110

[B35] Goh Y. J. Azcarate-Peril M. A. O’Flaherty S. Durmaz E. Valence F. Jardin J. (2009). Development and application of an upp-based counterselective gene replacement system for the study of the S-layer protein SlpX of *Lactobacillus acidophilus* NCFM. *Appl. Environ. Microbiol.* 75 3093–3105. 10.1128/AEM.02502-08 19304841 PMC2681627

[B36] Goh Y. J. Klaenhammer T. R. (2010). Functional roles of aggregation-promoting-like factor in stress tolerance and adherence of *Lactobacillus acidophilus* NCFM. *Appl. Environ. Microbiol.* 76 5005–5012. 10.1128/AEM.00030-10 20562289 PMC2916482

[B37] Golowczyc M. A. Mobili P. Garrote G. L. Abraham A. G. De Antoni G. L. (2007). Protective action of *Lactobacillus kefir* carrying S-layer protein against *Salmonella enterica* serovar Enteritidis. *Int. J. Food Microbiol.* 118 264–273. 10.1016/j.ijfoodmicro.2007.07.042 17719671

[B38] Grosu-Tudor S.-S. Brown L. Hebert E. M. Brezeanu A. Brinzan A. Fadda S. (2016). S-layer production by *Lactobacillus acidophilus* IBB 801 under environmental stress conditions. *Appl. Microbiol. Biotechnol.* 100 4573–4583. 10.1007/s00253-016-7355-5 26910041

[B39] Halttunen T. Collado M. C. El-Nezami H. Meriluoto J. Salminen S. (2008). Combining strains of lactic acid bacteria may reduce their toxin and heavy metal removal efficiency from aqueous solution. *Lett. Appl. Microbiol.* 46 160–165. 10.1111/j.1472-765X.2007.02276.x 18028332

[B40] Hennig C. Panak P. J. Reich T. Rossberg A. Raff J. Selenska-Pobell S. (2001). EXAFS investigation of uranium (VI) complexes formed at *Bacillus cereus* and *Bacillus sphaericus* surfaces. *Radiochim. Acta* 89 625–631.

[B41] Hirakata Y. Izumikawa K. Yamaguchi T. Igimi S. Furuya N. Maesaki S. (1998). Adherence to and penetration of human intestinal Caco-2 epithelial cell monolayers by *Pseudomonas aeruginosa*. *Infect. Immun.* 66 1748–1751. 9529107 10.1128/iai.66.4.1748-1751.1998 PMC108114

[B42] Hollmann A. Delfederico L. Glikmann G. De Antoni G. Semorile L. Disalvo E. A. (2007). Characterization of liposomes coated with S-layer proteins from lactobacilli. *Biochim. Biophys. Acta* 1768 393–400. 10.1016/j.bbamem.2006.09.009 17276386

[B43] Houwink A. L. (1953). A macromolecular mono-layer in the cell wall of *Spirillum* spec. *Biochim. Biophys. Acta* 10 360–366. 13058992 10.1016/0006-3002(53)90266-2

[B44] Hymes J. P. Johnson B. R. Barrangou R. Klaenhammer T. R. (2016). Functional analysis of an S-layer-associated fibronectin-binding protein in *Lactobacillus acidophilus* NCFM. *Appl. Environ. Microbiol.* 82 2676–2685. 10.1128/AEM.00024-16 26921419 PMC4836419

[B45] Hynönen U. Palva A. (2013). *Lactobacillus* surface layer proteins: structure, function and applications. *Appl. Microbiol. Biotechnol.* 97 5225–5243. 10.1007/s00253-013-4962-2 23677442 PMC3666127

[B46] Hynönen U. Westerlund-Wikström B. Palva A. Korhonen T. K. (2002). Identification by flagellum display of an epithelial cell- and fibronectin-binding function in the SlpA surface protein of *Lactobacillus brevis*. *J. Bacteriol.* 184 3360–3367. 12029053 10.1128/JB.184.12.3360-3367.2002 PMC135103

[B47] Ibrahim F. Halttunen T. Tahvonen R. Salminen S. (2006). Probiotic bacteria as potential detoxification tools: assessing their heavy metal binding isotherms. *Can. J. Microbiol.* 52 877–885. 17110980 10.1139/w06-043

[B48] Jankovic I. Ventura M. Meylan V. Rouvet M. Elli M. Zink R. (2003). Contribution of aggregation-promoting factor to maintenance of cell shape in *Lactobacillus gasseri* 4B2. *J. Bacteriol.* 185 3288–3296. 12754226 10.1128/JB.185.11.3288-3296.2003 PMC155393

[B49] Johansson M. E. V. Ambort D. Pelaseyed T. Schütte A. Gustafsson J. K. Ermund A. (2011). Composition and functional role of the mucus layers in the intestine. *Cell. Mol. Life Sci.* 68 3635–3641. 10.1007/s00018-011-0822-3 21947475 PMC11114784

[B50] Johnson B. Selle K. O’Flaherty S. Goh Y. J. Klaenhammer T. (2013). Identification of extracellular surface-layer associated proteins in *Lactobacillus acidophilus* NCFM. *Microbiology* 159 2269–2282. 10.1099/mic.0.070755-0 24002751 PMC3836491

[B51] Johnson B. R. Hymes J. Sanozky-Dawes R. Henriksen E. D. Barrangou R. Klaenhammer T. R. (2016). Conserved S-layer-associated proteins revealed by exoproteomic survey of S-layer-forming lactobacilli. *Appl. Environ. Microbiol.* 82 134–145. 10.1128/AEM.01968-15 26475115 PMC4702614

[B52] Johnson B. R. O’Flaherty S. Goh Y. J. Carroll I. Barrangou R. Klaenhammer T. R. (2017). The S-layer associated serine protease homolog PrtX impacts cell surface-mediated microbe-host interactions of *Lactobacillus acidophilus* NCFM. *Front. Microbiol.* 8:1185. 10.3389/fmicb.2017.01185 28713337 PMC5491966

[B53] Johnson-Henry K. C. Hagen K. E. Gordonpour M. Tompkins T. A. Sherman P. M. (2007). Surface-layer protein extracts from *Lactobacillus helveticus* inhibit enterohaemorrhagic *Escherichia coli* O157:H7 adhesion to epithelial cells. *Cell. Microbiol.* 9 356–367. 10.1111/j.1462-5822.2006.00791.x 16925785

[B54] Khaleghi M. Kasra R. (2012). “Effect of environmental stresses on S-Layer production in *Lactobacillus acidophilus* ATCC 4356” in *Advances in Applied Biotechnology*, ed. Petre M. (Rijeka: InTech).

[B55] Khaleghi M. Kermanshahi R. K. Yaghoobi M. M. Zarkesh-Esfahani S. H. Baghizadeh A. (2010). Assessment of bile salt effects on S-layer production, *slp* gene expression and some physicochemical properties of *Lactobacillus acidophilus* ATCC 4356. *J. Microbiol. Biotechnol.* 20 749–756. 20467248

[B56] Klingberg T. D. Pedersen M. H. Cencic A. Budde B. B. (2005). Application of Measurements of transepithelial electrical resistance of intestinal epithelial cell monolayers to evaluate probiotic activity. *Appl. Environ. Microbiol.* 71 7528–7530. 10.1128/AEM.71.11.7528-7530.2005 16269795 PMC1287686

[B57] Konstantinov S. R. Smidt H. de Vos W. M. Bruijns S. C. M. Singh S. K. Valence F. (2008). S-layer protein A of *Lactobacillus acidophilus* NCFM regulates immature dendritic cell and T cell functions. *Proc. Natl. Acad. Sci. U.S.A.* 105 19474–19479. 10.1073/pnas.0810305105 19047644 PMC2592362

[B58] Koval S. F. Murray R. G. (1984). The isolation of surface array proteins from bacteria. *Can. J. Biochem. Cell Biol. Rev. Can. Biochim. Biol. Cell.* 62 1181–1189. 10.1139/o84-152 6525568

[B59] Le Maréchal C. Peton V. Plé C. Vroland C. Jardin J. Briard-Bion V. (2015). Surface proteins of *Propionibacterium freudenreichii* are involved in its anti-inflammatory properties. *J. Proteomics* 113 447–461. 10.1016/j.jprot.2014.07.018 25150945

[B60] Lebeer S. Vanderleyden J. De Keersmaecker S. C. J. (2008). Genes and molecules of lactobacilli supporting probiotic action. *Microbiol. Mol. Biol. Rev.* 72 728–764. 10.1128/MMBR.00017-08 19052326 PMC2593565

[B61] Li P. Ye X. Wang Z. Yu Q. Yang Q. (2010). Effects of S-layer proteins from *Lactobacillus* against *Salmonella* typhimurium adhesion and invasion on Caco-2 cells. *Wei Sheng Wu Xue Bao* 50 1226–1231. 21090263

[B62] Li P. Yu Q. Ye X. Wang Z. Yang Q. (2011). *Lactobacillus* S-layer protein inhibition of *Salmonella*-induced reorganization of the cytoskeleton and activation of MAPK signalling pathways in Caco-2 cells. *Microbiol. Read. Engl.* 157 2639–2646. 10.1099/mic.0.049148-0 21700664

[B63] Lightfoot Y. L. Selle K. Yang T. Goh Y. J. Sahay B. Zadeh M. (2015). SIGNR3-dependent immune regulation by *Lactobacillus acidophilus* surface layer protein A in colitis. *EMBO J.* 34 881–895. 10.15252/embj.201490296 25666591 PMC4388597

[B64] Lin Y.-P. McDonough S. P. Sharma Y. Chang Y.-F. (2010). The terminal immunoglobulin-like repeats of LigA and LigB of *Leptospira* enhance their binding to gelatin binding domain of fibronectin and host cells. *PLoS One* 5:e11301. 10.1371/journal.pone.0011301 20585579 PMC2892007

[B65] Liu Z. Shen T. Chen H. Zhou Y. Zhang P. Ma Y. (2011a). Functional characterization of MIMP for its adhesion to the intestinal epithelium. *Front. Biosci.* 16:2106–2127. 21622165 10.2741/3842

[B66] Liu Z. Shen T. Zhang P. Ma Y. Qin H. (2011b). *Lactobacillus plantarum* surface layer adhesive protein protects intestinal epithelial cells against tight junction injury induced by enteropathogenic *Escherichia coli*. *Mol. Biol. Rep.* 38 3471–3480. 10.1007/s11033-010-0457-8 21086172

[B67] Lortal S. Rouault A. Cesselin B. Sleytr U. B. (1993). Paracrystalline surface layers of dairy propionibacteria. *Appl. Environ. Microbiol.* 59 2369–2374. 8304753 10.1128/aem.59.8.2369-2374.1993 PMC182293

[B68] Lortal S. Van Heijenoort J. Gruber K. Sleytr U. B. (1992). S-layer of *Lactobacillus helveticus* ATCC 12046: isolation chemical characterization and re-formation after extraction with lithium chloride. *J. Gen. Microbiol.* 138 611–618.

[B69] Maoret J. J. Font J. Augeron C. Codogno P. Bauvy C. Aubery M. (1989). A mucus-secreting human colonic cancer cell line. Purification and partial characterization of the secreted mucins. *Biochem. J.* 258 793–799. 10.1042/bj2580793 2658973 PMC1138434

[B70] Martínez M. G. Prado Acosta M. Candurra N. A. Ruzal S. M. (2012). S-layer proteins of *Lactobacillus acidophilus* inhibits JUNV infection. *Biochem. Biophys. Res. Commun.* 422 590–595. 10.1016/j.bbrc.2012.05.031 22595457 PMC7124250

[B71] Martínez-Maqueda D. Miralles B. Recio I. (2015). “HT29 cell line,” in *The Impact of Food Bioactives on Health*, eds Verhoeckx K. Cotter P. López-Expósito I. Kleiveland C. Lea T. Mackie A. (Cham: Springer International Publishing), 113–124. 10.1007/978-3-319-16104-4_11

[B72] Meng J. Zhu X. Gao S.-M. Zhang Q.-X. Sun Z. Lu R.-R. (2014). Characterization of surface layer proteins and its role in probiotic properties of three *Lactobacillus* strains. *Int. J. Biol. Macromol.* 65 110–114. 10.1016/j.ijbiomac.2014.01.024 24444879

[B73] Merroun M. L. Raff J. Rossberg A. Hennig C. Reich T. Selenska-Pobell S. (2005). Complexation of uranium by cells and S-layer sheets of *Bacillus sphaericus* JG-A12. *Appl. Environ. Microbiol.* 71 5532–5543. 10.1128/AEM.71.9.5532-5543.2005 16151146 PMC1214696

[B74] Messner P. Steiner K. Zarschler K. Schäffer C. (2008). S-layer nanoglycobiology of bacteria. *Carbohydr. Res.* 343 1934–1951. 10.1016/j.carres.2007.12.025 18336801 PMC4381302

[B75] Michon C. Langella P. Eijsink V. G. H. Mathiesen G. Chatel J. M. (2016). Display of recombinant proteins at the surface of lactic acid bacteria: strategies and applications. *Microb. Cell Fact.* 15:70. 10.1186/s12934-016-0468-9 27142045 PMC4855500

[B76] Mobili P. Gerbino E. Tymczyszyn E. Gómez-Zavaglia A. (2010). S-layers in lactobacilli: structural characteristics and putative role in surface and probiotic properties of whole bacteria. *Curr. Res. Technol. Educ. Top. Appl. Microbiol. Microb. Biotechnol.* 22 1224–1234.

[B77] Otte J.-M. Podolsky D. K. (2004). Functional modulation of enterocytes by gram-positive and gram-negative microorganisms. *Am. J. Physiol. Gastrointest. Liver Physiol.* 286 G613–G626. 10.1152/ajpgi.00341.2003 15010363

[B78] Prado Acosta M. Ruzal S. M. Cordo S. M. (2016). S-layer proteins from *Lactobacillus* sp. inhibit bacterial infection by blockage of DC-SIGN cell receptor. *Int. J. Biol. Macromol.* 92 998–1005. 10.1016/j.ijbiomac.2016.07.096 27498415

[B79] Pum D. Sleytr U. B. (2014). Reassembly of S-layer proteins. *Nanotechnology* 25:312001. 10.1088/0957-4484/25/31/312001 25030207

[B80] Qin H. Zhang Z. Hang X. Jiang Y. (2009). *L. plantarum* prevents enteroinvasive *Escherichia coli*-induced tight junction proteins changes in intestinal epithelial cells. *BMC Microbiol.* 9:63. 10.1186/1471-2180-9-63 19331693 PMC2674056

[B81] Rabah H. Rosa do Carmo F. L. Jan G. (2017). Dairy propionibacteria: versatile probiotics. *Microorganisms* 5:E24. 10.3390/microorganisms5020024 28505101 PMC5488095

[B82] Rong J. Zheng H. Liu M. Hu X. Wang T. Zhang X. (2015). Probiotic and anti-inflammatory attributes of an isolate *Lactobacillus helveticus* NS8 from Mongolian fermented koumiss. *BMC Microbiol.* 15:196. 10.1186/s12866-015-0525-2 26428623 PMC4591576

[B83] Rook G. Bäckhed F. Levin B. R. McFall-Ngai M. J. McLean A. R. (2017). Evolution, human-microbe interactions, and life history plasticity. *Lancet* 390 521–530. 10.1016/S0140-6736(17)30566-4 28792414

[B84] Rothfuss H. Lara J. C. Schmid A. K. Lidstrom M. E. (2006). Involvement of the S-layer proteins Hpi and SlpA in the maintenance of cell envelope integrity in *Deinococcus radiodurans* R1. *Microbiol. Read. Engl.* 152 2779–2787. 10.1099/mic.0.28971-0 16946272

[B85] Sanchez-Muñoz F. Dominguez-Lopez A. Yamamoto-Furusho J. K. (2008). Role of cytokines in inflammatory bowel disease. *World J. Gastroenterol.* 14 4280–4288. 10.3748/wjg.14.4280 18666314 PMC2731177

[B86] Sára M. Sleytr U. B. (1996). Crystalline bacterial cell surface layers (S-layers): from cell structure to biomimetics. *Prog. Biophys. Mol. Biol.* 65 83–111. 10.1016/S0079-6107(96)00007-7 9029942

[B87] Sára M. Sleytr U. B. (2000). S-Layer proteins. *J. Bacteriol.* 182 859–868. 10648507 10.1128/jb.182.4.859-868.2000 PMC94357

[B88] Schuster B. Sleytr U. B. (2015). Relevance of glycosylation of S-layer proteins for cell surface properties. *Acta Biomater.* 19 149–157. 10.1016/j.actbio.2015.03.020 25818946 PMC4414373

[B89] Sengupta R. Altermann E. Anderson R. C. McNabb W. C. Moughan P. J. Roy N. C. (2013). The role of cell surface architecture of lactobacilli in host-microbe interactions in the gastrointestinal tract. *Mediators Inflamm.* 2013:237921. 10.1155/2013/237921 23576850 PMC3610365

[B90] Sleytr U. B. (1997). I. Basic and applied S-layer research: an overview. *FEMS Microbiol. Rev.* 20 5–12. 10.1111/j.1574-6976.1997.tb00301.x 29312266

[B91] Sleytr U. B. Beveridge T. J. (1999). Bacterial S-layers. *Trends Microbiol.* 7 253–260. 10366863 10.1016/s0966-842x(99)01513-9

[B92] Sleytr U. B. Messner P. (1988). Crystalline surface layers in procaryotes. *J. Bacteriol.* 170 2891–2897. 3290191 10.1128/jb.170.7.2891-2897.1988 PMC211226

[B93] Sleytr U. B. Sára M. Pum D. Schuster B. (2001). Characterization and use of crystalline bacterial cell surface layers. *Prog. Surf. Sci.* 68 231–278. 10.1016/S0079-6816(01)00008-9

[B94] Sleytr U. B. Schuster B. Egelseer E.-M. Pum D. (2014). S-layers: principles and applications. *FEMS Microbiol. Rev.* 38 823–864. 10.1111/1574-6976.12063 24483139 PMC4232325

[B95] Sleytr U. B. Schuster B. Egelseer E. M. Pum D. Horejs C. M. Tscheliessnig R. (2011). Nanobiotechnology with S-layer proteins as building blocks. *Prog. Mol. Biol. Transl. Sci.* 103 277–352. 10.1016/B978-0-12-415906-8.00003-0 21999999

[B96] Smit E. Oling F. Demel R. Martinez B. Pouwels P. H. (2001). The S-layer protein of *Lactobacillus acidophilus* ATCC 4356: identification and characterization of domains responsible for S-protein assembly and cell wall binding. *J. Mol. Biol.* 305 245–257. 10.1006/jmbi.2000.4258 11124903

[B97] Syngai G. G. Gopi R. Bharali R. Dey S. Lakshmanan G. M. A. Ahmed G. (2016). Probiotics - the versatile functional food ingredients. *J. Food Sci. Technol.* 53 921–933. 10.1007/s13197-015-2011-0 27162372 PMC4837740

[B98] Taverniti V. Stuknyte M. Minuzzo M. Arioli S. De Noni I. Scabiosi C. (2013). S-layer protein mediates the stimulatory effect of *Lactobacillus helveticus* MIMLh5 on innate immunity. *Appl. Environ. Microbiol.* 79 1221–1231. 10.1128/AEM.03056-12 23220964 PMC3568609

[B99] Uroić K. Novak J. Hynönen U. Pietilä T. E. Leboš Pavunc A. Kant R. (2016). The role of S-layer in adhesive and immunomodulating properties of probiotic starter culture *Lactobacillus brevis* D6 isolated from artisanal smoked fresh cheese. *Food Sci. Technol.* 69 623–632. 10.1016/j.lwt.2016.02.013

[B100] Velásquez L. Dussan J. (2009). Biosorption and bioaccumulation of heavy metals on dead and living biomass of *Bacillus sphaericus*. *J. Hazard. Mater.* 167 713–716. 10.1016/j.jhazmat.2009.01.044 19201532

[B101] Velasquez-Manoff M. (2015). Gut microbiome: the peacekeepers. *Nature* 518 S3–S11. 10.1038/518S3a 25715278

[B102] Vélez M. P. De Keersmaecker S. C. J. Vanderleyden J. (2007). Adherence factors of *Lactobacillus* in the human gastrointestinal tract. *FEMS Microbiol. Lett.* 276 140–148. 10.1111/j.1574-6968.2007.00908.x 17888009

[B103] Ventura M. Jankovic I. Walker D. C. Pridmore R. D. Zink R. (2002). Identification and characterization of novel surface proteins in *Lactobacillus johnsonii* and *Lactobacillus gasseri*. *Appl. Environ. Microbiol.* 68 6172–6181. 12450842 10.1128/AEM.68.12.6172-6181.2002 PMC134427

[B104] Vesterlund S. Paltta J. Karp M. Ouwehand A. C. (2005). Measurement of bacterial adhesion-in vitro evaluation of different methods. *J. Microbiol. Methods* 60 225–233. 10.1016/j.mimet.2004.09.013 15590097

[B105] Vindigni S. M. Zisman T. L. Suskind D. L. Damman C. J. (2016). The intestinal microbiome, barrier function, and immune system in inflammatory bowel disease: a tripartite pathophysiological circuit with implications for new therapeutic directions. *Ther. Adv. Gastroenterol.* 9 606–625. 10.1177/1756283X16644242 27366227 PMC4913337

[B106] Wang R. Jiang L. Zhang M. Zhao L. Hao Y. Guo H. (2017). The Adhesion of *Lactobacillus salivarius* REN to a Human intestinal epithelial cell line requires S-layer proteins. *Sci. Rep.* 7:44029. 10.1038/srep44029 28281568 PMC5345100

[B107] Waśko A. Polak-Berecka M. Paduch R. Jóźwiak K. (2014). The effect of moonlighting proteins on the adhesion and aggregation ability of *Lactobacillus helveticus*. *Anaerobe* 30C, 161–168. 10.1016/j.anaerobe.2014.10.002 25445202

[B108] Zaneveld J. Turnbaugh P. J. Lozupone C. Ley R. E. Hamady M. Gordon J. I. (2008). Host-bacterial coevolution and the search for new drug targets. *Curr. Opin. Chem. Biol.* 12 109–114. 10.1016/j.cbpa.2008.01.015 18280814 PMC2348432

[B109] Zhang W. Wang H. Liu J. Zhao Y. Gao K. Zhang J. (2013). Adhesive ability means inhibition activities for *Lactobacillus* against pathogens and S-layer protein plays an important role in adhesion. *Anaerobe* 22 97–103. 10.1016/j.anaerobe.2013.06.005 23792230

[B110] Zhang Y. Xiang X. Lu Q. Zhang L. Ma F. Wang L. (2016). Adhesions of extracellular surface-layer associated proteins in *Lactobacillus* M5-L and Q8-L. *J. Dairy Sci.* 99 1011–1018. 10.3168/jds.2015-10020 26709174

